# Circular RNA hsa-circ-0001030 suppresses proliferation and cisplatin tolerance in TSCC via interaction with PKM2

**DOI:** 10.20517/cdr.2025.200

**Published:** 2026-01-07

**Authors:** Haojie Yang, Yingzhe Yan, Zicong Tan, Xiaoying Xu, Kang Chen, Qin Li, Ning Liufu, Fengtao Ji

**Affiliations:** ^1^Department of Anesthesia, Sun Yat-sen Memorial Hospital, Sun Yat-sen University, Guangzhou 510000, Guangdong, China.; ^2^Guangdong Provincial Key Laboratory of Malignant Tumor Epigenetics and Gene Regulation, Guangdong-Hong Kong Joint Laboratory for RNA Medicine, Medical Research Center, Sun Yat-sen Memorial Hospital, Sun Yat-sen University, Guangzhou 510000, Guangdong, China.; ^3^Department of Pathology, Sun Yat-Sen Memorial Hospital, Sun Yat-Sen University, Guangzhou 510000, Guangdong, China.; ^#^These authors contributed equally to this work and share first authorship.

**Keywords:** Tongue squamous cell carcinoma, circRNA, hsa-circ-0001030, PKM2, glycolysis, cisplatin resistance, metabolic reprogramming

## Abstract

**Aim:** Cisplatin resistance remains a major obstacle to the effective treatment of tongue squamous cell carcinoma (TSCC). This study is dedicated to elucidating the role and mechanism of circular RNA (circRNA) hsa-circ-0001030 in modulating cisplatin sensitivity and metabolic reprogramming in TSCC.

**Methods:** CircRNA sequencing, quantitative polymerase chain reaction, and RNA fluorescence *in situ* hybridization were used to test hsa-circ-0001030 expression in TSCC tissues and cell lines. Gain-of-function assays (colony formation, cell counting kit-8, Transwell assay, and xenograft models) were conducted to evaluate proliferation, invasion, and cisplatin response. Mechanistic studies, including RNA pull-down, RNA-binding protein immunoprecipitation, and western blotting, were performed to identify pyruvate kinase M2 (PKM2) as a binding partner of hsa-circ-0001030 and to assess glycolytic activity, glucose uptake, and lactate production.

**Results:** Hsa-circ-0001030 was markedly downregulated in TSCC and cisplatin-resistant cells. Overexpression of hsa-circ-0001030 suppressed tumor growth, migration, and glycolytic flux, while enhancing cisplatin sensitivity both *in vitro* and *in vivo*. Mechanistically, hsa-circ-0001030 directly bound to PKM2 at nucleotides 138-169, inhibited PKM2 enzymatic activity, restraining tetramer formation and increased tyrosine 105 (Tyr105) phosphorylation and thereby blocking PKM2-driven glycolysis. Clinically, low hsa-circ-0001030 expression correlated with advanced tumor-node-metastasis stage, poor differentiation, and unsatisfying prognosis in TSCC patients.

**Conclusion:** Hsa-circ-0001030 acted as a tumor-suppressive circRNA that might depress PKM2-dependent metabolic reprogramming and cisplatin resistance in TSCC, highlighting its potential as a prognostic biomarker and therapeutic target for overcoming chemoresistance.

## INTRODUCTION

Tongue squamous cell carcinoma (TSCC), one of the most common subtypes of head and neck squamous cell carcinoma (HNSCC), remains among the most aggressive oral malignancies worldwide^[1]^. Despite advances in surgery, radiotherapy, and chemotherapy, the prognosis for patients with advanced or recurrent TSCC remains poor due to early lymph node metastasis and the development of chemoresistance^[2]^. Among available chemotherapeutic agents, cisplatin remains the standard first-line drug for TSCC; however, intrinsic or acquired resistance frequently limits its efficacy and leads to treatment failure^[3]^. Therefore, elucidating the molecular mechanisms underlying cisplatin resistance is of great significance for improving therapeutic outcomes in TSCC.

Recent studies have highlighted the crucial role of metabolic reprogramming in tumor progression and drug resistance^[4]^. Cancer cells preferentially rely on aerobic glycolysis, known as the Warburg effect, to meet their increased bioenergetic and biosynthetic demands^[5]^. Pyruvate kinase M2 (PKM2), a key rate-limiting enzyme in glycolysis, plays a decisive role in this process by catalyzing the conversion of phosphoenolpyruvate to pyruvate^[6,7]^. Beyond its canonical metabolic function, PKM2 also acts as a protein kinase and transcriptional coactivator that promotes tumor cell growth, survival, and therapy resistance. Aberrant activation or nuclear translocation of PKM2 has been linked to poor response to chemotherapy in various cancers, including HNSCC, suggesting that PKM2-mediated glycolytic reprogramming contributes to cisplatin resistance^[8]^.

Circular RNAs (circRNAs), known as a unique class of covalently closed noncoding RNAs, emerged as important regulators of gene expression and signaling in cancer^[9]^. Their resistance to exonuclease degradation and tissue-specific expression patterns make them stable and functionally versatile molecules. Increasing evidence indicates that circRNAs can modulate tumor metabolism and chemoresistance through interactions with proteins or by functioning as competing endogenous RNAs^[10]^. For example, circMAT2B [a circular RNA generated from the methionine adenosyltransferase 2 beta (*MAT2B*) gene] enhances tumor progression and glycolysis in hepatocellular carcinoma (HCC) and mechanically activates the miR-338-3p/PKM2 axis, while circP4HB [a circular RNA generated from the prolyl 4-hydroxylase subunit beta (*P4HB*) gene] promotes PKM2 tetramer formation and glycolytic flux in lung adenocarcinoma^[11]^. However, the role of circRNAs in metabolic regulation and cisplatin resistance in TSCC remains largely unexplored.

In this study, hsa-circ-0001030 was recognized as a significantly downregulated circRNA in TSCC tissues and cell lines. Functional assays revealed that hsa-circ-0001030 suppresses proliferation, invasion, and glycolytic metabolism, while enhancing cisplatin sensitivity. Mechanistically, hsa-circ-0001030 directly binds to PKM2, inhibits its enzymatic activity, and attenuates glycolytic reprogramming in TSCC cells. Clinically, decreased hsa-circ-0001030 expression is considered to be related to advanced tumor stage and poor prognosis. These findings uncover a novel regulatory mechanism linking circRNA-mediated metabolic control to cisplatin resistance in TSCC and highlight hsa-circ-0001030 as a potential therapeutic target for overcoming chemoresistance.

## METHODS

### Specimens and cell lines

A total of 88 primary TSCC tissues and 11 cisplatin-resistant TSCC specimens were collected from patients with surgical resection and without receiving radiotherapy or chemotherapy prior to surgery at the Department of Oral and Maxillofacial Surgery, Sun Yat-sen Memorial Hospital, Sun Yat-sen University (Guangzhou, China) between 2015 and 2020. All specimens were pathologically confirmed as TSCC by two independent pathologists. Written informed consent was obtained from all participants, and the approval of the protocol was obtained from the Ethics Committee of Sun Yat-sen Memorial Hospital (Approval No. [SYSKY-2025-379-01]), in accordance with the principles of the Declaration of Helsinki. Normal oral keratinocytes (NOK) were acquired from the Procell Life Science Technology (Wuhan, China). Cell lines of human TSCC (Cal27, SCC9, Tca8113, OSCC3, HSC6, and Cal33) were acquired from the American Type Culture Collection (ATCC, Manassas, VA, USA). All cell lines used in this study were authenticated by short tandem repeat (STR) profiling and confirmed to be free of Mycoplasma contamination. Cells were cultured in Dulbecco’s modified Eagle’s medium (DMEM) added with 10% fetal bovine serum (FBS; Gibco, USA) and 1% penicillin–streptomycin, and maintained at 37 °C in a humidified atmosphere containing 5% CO_2_. Cisplatin-resistant sublines [cisplatin-resistant Cal27 cells (Ca727R), cisplatin-resistant SCC9 cells (SCC9R), and cisplatin-resistant Tca8113 cells (Tca8113R)] were established by continuously exposing the parental cells to gradually increasing concentrations of cisplatin (Sigma–Aldrich, USA) ranging from 0.1 to 10 μM over a period of more than six months, following the protocol described in our previously published study^[12]^. The resistant phenotype was validated using cell viability and half-maximal inhibitory concentration (IC_50_) assays. Parental cell lines were maintained in cisplatin-free medium, whereas resistant sublines were cultured in medium containing 1 μM cisplatin for maintenance, with the drug withdrawn for at least two passages before subsequent experiments. For circRNA overexpression, lentiviral vectors encoding hsa-circ-0001030 or an empty vector control were purchased from GenePharma (Shanghai, China). TSCC cells were transduced with 10 μg/mL Polybrene and screened with 2 μg/mL puromycin for seven continuous days. Stable luciferase-tagged cell lines were generated using 300 μg/mL neomycin and were used for *in vivo* imaging assays.

### Arraystar circRNA microarray analysis and Sanger sequencing validation

To identify differentially expressed circRNAs in TSCC, total RNA from paired tumor and adjacent normal tissues was extracted by TRIzol reagent (Thermo Fisher Scientific, USA). RNA was quantified using a NanoDrop 2000 spectrophotometer (Thermo Fisher Scientific, USA), and RNA integrity was confirmed by agarose gel electrophoresis. circRNA expression profiling was conducted using the Arraystar Human circRNA Microarray (8 × 15 K; Arraystar Inc., Rockville, MD, USA). In accordance with the manufacturer’s protocols, sample labeling, hybridization, and scanning were performed. The raw data were normalized and analyzed using R software (version 4.2.0). Differentially expressed circRNAs were identified with thresholds of |log_2_FC| > 1 and *P* < 0.05, where FC refers to fold change. Candidate circRNAs were further validated by polymerase chain reaction (PCR). CircRNAs were amplified using divergent primers specific to the back-splice junctions. PCR products were validated by Sanger sequencing (Sangon Biotech, Shanghai, China) to verify the circular junction sequences.

### RNase R treatment

To test the circular structure and exonuclease resistance of hsa-circ-0001030, total RNA (10 μg) extracted from TSCC cells was treated with RNase R (3 U/μg RNA; GeneSeed Biotech, Guangzhou, China) in the presence of Reaction Buffer supplied by the manufacturer. After incubating the mixture at 37 °C for 30 min, enzyme inactivation was performed at 70 °C for 10 min. An equal amount of untreated RNA served as the control. Phenol–chloroform extraction was used to purify the remaining RNA. RNA was quantified with a NanoDrop 2000 spectrophotometer (Thermo Fisher Scientific, USA). Digested samples and control samples were both subsequently subjected to quantitative real-time PCR (qRT-PCR) to score the relative stability of hsa-circ-0001030 and its linear parental messenger RNA (mRNA; EXOC6B). The main experimental steps are the same as the protocol described in our previously published study^[12]^.

### Assessment of RNA stability using actinomycin D

Comparing the stability of hsa-circ-0001030 and its linear transcript EXOC6B mRNA, TSCC cells in 6-well plates were seeded at a density of 5 × 10^5^ cells. Cells were cultured at 37 °C overnight in a humidified atmosphere containing 5% CO_2_. Seeded cells were then handled with Actinomycin D (Streptomyces antibiotic D, 2 μg/mL; MedChemExpress, Shanghai, China) to inhibit *de novo* RNA transcription. An equal volume of dimethyl sulfoxide (DMSO) was administered to the control group. Total RNA was extracted at 0, 2, 4, 8, 12, and 24 h after treatment using TRIzol reagent (Thermo Fisher Scientific, USA). The relative RNA abundance of hsa-circ-0001030 and EXOC6B mRNA at each time point was quantified by qRT-PCR, normalizing to glyceraldehyde-3-phosphate dehydrogenase (GAPDH) expression. RNA decay curves were plotted to assess transcript stability, and all experiments were performed in triplicate.

### RNA nucleocytoplasmic fractionation

To determine the subcellular localization of hsa-circ-0001030, cytoplasmic and nuclear RNA fractions were isolated using the PARIS™ Kit (Thermo Fisher Scientific, USA) according to the manufacturer’s instructions. Briefly, TSCC cells were washed twice with ice-cold phosphate-buffered saline (PBS) and lysed in Cell Fractionation Buffer on ice for 10 min. The lysate was centrifuged at 500 × *g* for 5 min at 4 °C, and the supernatant containing the cytoplasmic fraction was carefully collected. The remaining nuclear pellet was resuspended in Cell Disruption Buffer and the nuclear fraction was then obtained. RNA from both fractions was extracted using Lysis/Binding Solution, followed by purification according to the kit protocol. A NanoDrop 2000 spectrophotometer (Thermo Fisher Scientific, USA) was used for assessing RNA concentration and purity. The relative abundance of hsa-circ-0001030 in nuclear and cytoplasmic fractions was quantified by qRT-PCR. GAPDH and U6 served as cytoplasmic and nuclear internal controls, respectively. Each experiment was performed in triplicate.

### qRT-PCR

qRT-PCR was performed to quantify the expression levels of hsa-circ-0001030, its linear host gene *EXOC6B*, and other target genes. Following the manufacturer’s instructions, TRIzol reagent (Thermo Fisher Scientific, USA) was used to extract total RNA from cultured cells or tissue samples. RNA concentration and purity were verified by a NanoDrop 2000 spectrophotometer (Thermo Fisher Scientific, USA). Samples meeting standards of A260/A280 ratios between 1.8 and 2.1 were screened for further analyses. Evo M-MLV Reverse Transcription Kit (Takara, Dalian, China) and random hexamer primers were used in reverse transcription, and 1 μg of total RNA was converted into complementary DNA (cDNA). On the platform of QuantStudio™ 6 Flex Real-Time PCR System (Applied Biosystems, USA), qRT-PCR was conducted with Hieff® qRT-PCR SYBR Green Master Mix (Takara, Dalian, China). Each 20 μL reaction contained 2 μL of diluted cDNA, 0.5 μL of each primer (10 μM), 7 μL of nuclease-free water, and 10 μL SYBR Green Mix. The thermocycling was performed at 95 °C for 5 min, followed by 40 cycles of 95 °C for 10 s and 60 °C for 30 s. All reactions were triplicate-repeated, and GAPDH served as a normalizing control. Relative expression levels between RNAs were calculated with 2^-ΔΔCt^ method. Supplementary Table 1 listed primer sequences mentioned in this study.

### Western blot analysis

Expression and phosphorylation levels of PKM2 and other related proteins were all determined by western blotting. Cells were lysed in radio immunoprecipitation assay lysis buffer (RIPA buffer) (Beyotime, Shanghai, China), and added with protease and phosphatase inhibitor cocktail (Roche, Switzerland) on ice for 30 min. After centrifuging the lysates at 12,000 × *g* for 10 min at 4 °C and collecting the supernatants, the bicinchoninic acid (BCA) Protein Assay Kit (Thermo Fisher Scientific, USA) was used to test protein concentrations. Twenty micrograms of protein were separated by 8%-12% sodium dodecyl sulfate–polyacrylamide gel electrophoresis (SDS–PAGE) and transferred onto polyvinylidene difluoride (PVDF) membranes (Millipore, USA). Blocking the membranes with 5% non-fat dry milk in TBST buffer (Tris-buffered saline with 0.1% Tween-20) for 1 h at room temperature, membranes were incubated at 4 °C overnight with the primary antibodies, including PKM2 (1:1,000, Cell Signaling Technology, USA), phosphorylated pyruvate kinase M2 at tyrosine 105 [p-PKM2 (Tyr105), 1:1,000, Cell Signaling Technology], p-PKM2 (Ser37, 1:1,000, Biorbyt, UK), GAPDH (1:1,000, Cell Signaling Technology), and β-Tubulin (1:1,000, Cell Signaling Technology). After triplicate washing with TBST, membranes were incubated with secondary antibody (1:5,000, Abcam, UK) [Horseradish peroxidase (HRP)-conjugated goat anti-rabbit] for 1 h at room temperature. The Enhanced chemiluminescence (ECL) detection system (Thermo Fisher Scientific, USA) and ChemiDoc™ Imaging System (Bio-Rad, USA) could visualize and image the protein bands. Band intensities were quantified using ImageJ software [National Institutes of Health (NIH), USA], and protein expression levels were normalized to GAPDH or β-Tubulin.

### Detection of PKM2 tetramer formation

To assess the oligomeric status of PKM2, cells were harvested and lysed in non-denaturing lysis buffer [20 mM Tris-HCl, pH 7.4, 150 mM NaCl, 1 mM ethylenediaminetetraacetic acid (EDTA), and 1% Nonidet P-40 (NP-40)] on ice. Equal amounts of total protein were incubated with 0.025% glutaraldehyde (Sigma) for 10 min at room temperature to cross-link PKM2 complexes, and the reaction was then quenched with 1 M Tris-HCl (pH 8.0) for 5 min. The samples were mixed with loading buffer, denatured at 95 °C for 5 min, and separated by SDS–PAGE, followed by Western blot analysis as described above. Non-cross-linked lysates were analyzed in parallel to determine total PKM2 levels.

### Plate colony formation assay

To evaluate the clonogenic capacity of TSCC cells, colony-formation assays were performed. Briefly, 1 × 10^3^ cells were seeded into six-well plates and cultured under standard conditions for 10-14 days until visible colonies appeared. The medium was replaced every 3 days. Cells were washed twice with PBS gently, fixed with 4% paraformaldehyde for 15 min at room temperature, and stained with 0.1% crystal violet solution (Beyotime, Shanghai, China) for 20 min. Plates were then rinsed with tap water and air-dried. Only colonies containing more than 50 cells were counted manually under a stereomicroscope. Each experiment was repeated in triplicate, and the mean ± standard deviation (SD) was used for statistical analysis.

### Transwell migration and invasion assays

To assess the migration and invasion capacities of cisplatin-resistant TSCC cells and their parental controls, Transwell assays were performed. For migration assays, 2 × 10^5^ Cal27 or Cal27R cells and 8 × 10^4^ Tca8113 or Tca8113R cells were suspended in 700 μL serum-free DMEM. Suspended cells were seeded into a 24-well Transwell insert (8-μm pore size; Corning, USA) in the upper chamber. For invasion assays, Matrigel (Corning, USA) was used to pre-coat the upper chambers according to the manufacturer’s protocol. DMEM containing 10% FBS served as a chemoattractant, and the lower chambers were filled with it. Cells were treated with cisplatin (5 μM) and/or the PKM2 activator tetramer enhancer of pyruvate kinase M2-46 (TEPP-46, 20 μM) as indicated. Cal27/Cal27R cells were incubated for 36 h. Tca8113/Tca8113R cells were incubated for 48 h at 37 °C in 5% CO_2_. After incubation, non-migrated cells were removed from the upper surface of the membrane using a cotton swab. Methanol was used to fix cells on the lower surface for 15 min. Cells were then stained with 0.1% crystal violet (Beyotime, Shanghai, China) for 20 min, washed with PBS, and air-dried. Migrated or invaded cells were photographed using a Leica DMi8 inverted microscope (Leica Microsystems, Germany), and five random fields were counted per insert. Each experiment was conducted in triplicate. The main experimental steps are the same as the protocol described in our previously published study^[12]^.

### Cell viability assay (CCK-8)

Cell counting kit-8 (CCK-8; Dojindo Laboratories, Japan) was used to determine cell viability according to the manufacturer’s instructions. Briefly, TSCC cells in the logarithmic growth phase were seeded at a density of 4 × 10^3^ cells per well in 96-well plates and cultured overnight to achieve attachment. Cells were then exposed to cisplatin at various concentrations (0, 1, 2.5, 5, 10, 20, and 40 μM) for 24 h. For each well, 10 μL of CCK-8 reagent was added, followed by incubation for 2 h at 37 °C. A microplate reader (Thermo Fisher Scientific, USA) was used to measure the absorbance at 450 nm. Cell viability (%) was calculated according to the relative absorbance between treated wells and untreated controls. The IC_50_ was determined using nonlinear regression analysis with GraphPad Prism 8.0 (GraphPad Software, USA). Each experiment was independently repeated three times.

### RNA pull-down assay

RNA pull-down assays were performed to identify proteins or microRNAs (miRNAs) interacting with hsa-circ-0001030. Biotin-labeled sense and antisense probes specific to hsa-circ-0001030 were designed and synthesized by RiboBio Co., Ltd. (Guangzhou, China). TSCC cells were lysed in lysis buffer (Thermo Fisher Scientific, USA) containing protease and RNase inhibitors on ice for 30 min, and the supernatant was collected by centrifugation at 12,000 × *g* for 10 min at 4 °C. Equal amounts of cell lysate were incubated with biotin-labeled probes (100 pmol) with gentle rotation at 4 °C overnight. The RNA–protein or RNA–miRNA complexes were captured using streptavidin-coated magnetic beads (Invitrogen, USA) for 2 h at 4 °C. After three washes with wash buffer (150 mM NaCl, 10 mM Tris–HCl, pH 7.5, 0.1% NP-40), the bound complexes were eluted using biotin elution buffer or by heating at 95 °C for 5 min. The retrieved RNA was purified using TRIzol reagent (Thermo Fisher Scientific, USA) and analyzed by qRT-PCR to detect interacting miRNAs. Bound proteins were subjected to western blotting to confirm interactions between protein and RNA. Each experiment was repeated three times independently.

### RNA-binding protein immunoprecipitation assay

RNA-binding protein immunoprecipitation (RIP) assays were conducted to determine the association between hsa-circ-0001030, miRNAs, and the RNA-binding protein Ago2. Following the manufacturer’s protocol, the experiments were performed using the Magna RIP™ RNA-Binding Protein Immunoprecipitation Kit (Millipore, USA). TSCC cells were washed twice with ice-cold PBS and lysed in RIP lysis buffer supplemented with protease and RNase inhibitors. The lysates were centrifuged at 12,000 × *g* for 10 min at 4 °C. The subsequent supernatants were incubated at 4 °C overnight with magnetic beads pre-coated with anti-Ago2 antibody (Abcam, UK) or normal rabbit immunoglobulin G (IgG, Millipore, USA) as a negative control. After incubation, six washes for the beads were performed with RIP wash buffer, and the RNA–protein complexes were eluted. The bound RNA was purified using TRIzol reagent (Thermo Fisher Scientific, USA) and subjected to qRT-PCR analysis to detect hsa-circ-0001030 and associated miRNAs. Each assay was repeated three times independently. The main experimental steps are the same as the protocol described in our previously published study^[12]^.

### Measurement of PKM2 enzymatic activity, glucose consumption, and lactate production

To evaluate the glycolytic activity regulated by hsa-circ-0001030, PKM2 enzymatic activity, glucose consumption, and lactate production, commercial assay kits were used following the manufacturers’ protocols. PKM2 activity was measured using the Pyruvate Kinase Activity Assay Kit (Solarbio Life Sciences, Beijing, China). Briefly, cell lysates were prepared according to the kit instructions, and the enzymatic reaction was initiated by the substrate solution. The absorbance was recorded at 340 nm using a microplate reader (Thermo Fisher Scientific, USA), and PKM2 activity was normalized to total protein concentration determined by the BCA Protein Assay Kit (Thermo Fisher Scientific, USA). The Glucose Assay Kit and Lactate Assay Kit (Nanjing Jiancheng Bioengineering Institute, Nanjing, China) were used to measure the concentrations of glucose and lactate in cell culture supernatants, respectively. Cells were cultured under standard conditions for 24 h. Collected supernatants were centrifuged at 3,000 × *g* for 10 min, and then subjected to colorimetric analysis at 505 nm (glucose) and 530 nm (lactate). The results were described as relative levels normalized to cell number or total protein content. Each experiment was performed in triplicate.

### MTT cell proliferation assay

Cell proliferation was evaluated using the 3-(4,5-dimethylthiazol-2-yl)-2,5-diphenyltetrazolium bromide (MTT) assay following the manufacturer’s instructions. Briefly, cells were seeded in 96-well plates at a density of 2 × 10^3^ cells per well and cultured for 1-5 days under standard conditions (37 °C, 5% CO_2_). At the indicated time points, 20 μL of MTT solution (5 mg/mL; Solarbio Life Sciences, Beijing, China) was added to each well, and plates were incubated for 4 h at 37 °C to allow the formation of formazan crystals. After incubation, the supernatant was gently aspirated, and 150 μL of DMSO (Sigma-Aldrich, USA) was added to dissolve the crystals completely by shaking at room temperature for 10 min. The absorbance at 490 nm was recorded using a microplate reader (Thermo Fisher Scientific, USA). With three independent experiments, cell viability was expressed as the mean optical density (OD_490_).

### Gene ontology and gene set enrichment analysis

To explore the potential biological significances and downstream pathways associated with hsa-circ-0001030, differentially expressed genes (DEGs) were identified using the “limma” package in R software (version 4.2.0). Gene ontology (GO) enrichment analysis was performed to categorize DEGs into biological process (BP), molecular function (MF), and cellular component (CC) terms using the “clusterProfiler” package (version 4.8.0). Enrichment results were visualized with the “ggplot2” and “enrichplot” packages. Gene set enrichment analysis (GSEA) was conducted using the Molecular Signatures Database (MSigDB v7.5.1) as the reference. The enrichment score was calculated by a permutation-based algorithm (1,000 permutations), and pathways with |normalized enrichment score (NES)| > 1, nominal *P* < 0.05, and false discovery rate (FDR) < 0.25 were considered significantly enriched Functional annotation was further integrated with Kyoto Encyclopedia of Genes and Genomes (KEGG) analysis to identify key signaling pathways potentially involved in PKM2-related metabolic regulation in TSCC. The main experimental steps are the same as the protocol described in our previously published study^[12]^.

### Hematoxylin–eosin staining

Hematoxylin–eosin (HE) staining was performed to evaluate the histopathological morphology of TSCC tissues. Paraffin-embedded tissue specimens were cut into 4-μm-thick sections. After deparaffinizing in xylene, sections were rehydrated through a graded series of ethanol (100%, 95%, 80%) to distilled water. Subsequent staining was performed with hematoxylin for 5 min and eosin for 2 min, followed by dehydration in graded ethanol, clearing in xylene, and mounting with neutral resin. Histological structures, including tumor cell morphology, nuclear atypia, and stromal features, were observed using a Leica DM2500 light microscope (Leica Microsystems, Germany). Representative images were captured using Leica Application Suite X software.

### RNA fluorescence *in situ* hybridization

To determine the subcellular localization of hsa-circ-0001030 in TSCC cells, RNA fluorescence *in situ* hybridization (FISH) was performed. A cyanine 3 (Cy3)-labeled probe specifically targeting the back-splice junction of hsa-circ-0001030 was designed and synthesized by GenePharma (Shanghai, China). TSCC cells were seeded in 24-well plates on sterile glass coverslips and cultured overnight. Cells were fixed with 4% paraformaldehyde at room temperature for 15 min, permeabilized with 0.5% Triton X-100 for 5 min, and prehybridized for 30 min in hybridization buffer at 37 °C. The Cy3-labeled probe (2 μM) was then hybridized with the cells at 37 °C overnight in a humidified chamber. The hybridized slides were washed with 2× SSC buffer (saline–sodium citrate) three times to remove unbound probes. Nuclei were counterstained with 4′,6-diamidino-2-phenylindole (DAPI; Sigma-Aldrich, USA) for 5 min, and coverslips were mounted using anti-fade mounting medium (Vector Laboratories, USA). Fluorescence signals were visualized and imaged using a Zeiss LSM 880 confocal fluorescence microscope (Carl Zeiss, Germany). All experiments were performed in triplicate, and signal localization was analyzed using ImageJ software (NIH, USA).

### *In vivo* experiments

*In vivo* studies were conducted to evaluate the effects of hsa-circ-0001030 on tumor growth, metastasis, glycolytic response, and cisplatin sensitivity in TSCC. All procedures were approved by the Animal Ethics Committee of Sun Yat-sen Memorial Hospital, Sun Yat-sen University and complied with institutional guidelines. Male BALB/c nude mice, ~3 weeks old, were purchased from GemPharmatech (Jiangsu, China) and housed under SPF conditions with ad libitum access to food and water. The sample size of the mice for every group of every model was 5 (*n* = 5).

#### Orthotopic tongue tumor model

Cal27 cells (in a total volume of 25 μL PBS mixed with 25 μL Matrigel, Corning, USA, at a density of 5 × 10^6^) were inoculated into the mid-left tongue. One week later, mice were anesthetized and injected with D-luciferin (15 mg/mL; Yeasen, Shanghai, China) intraperitoneally for bioluminescence imaging on an IVIS-200 system; imaging was repeated at two weeks prior to euthanasia, and tongue tissues were collected for analysis.

#### Experimental lung metastasis model

To establish lung colonization, cells suspended in 200 μL PBS at a density of 2 × 10^5^ were injected via the tail vein. At day 28, mice received intraperitoneal D-luciferin (15 mg/mL) and underwent *in vivo* imaging (IVIS-200). Lungs were harvested post-euthanasia, 4% paraformaldehyde fixed, paraffin-embedded, and processed for downstream assays.

#### Subcutaneous xenograft with cisplatin treatment

Cal27R cells transfected with hsa-circ-0001030 or vector (5 × 10^6^ cells/mouse) stably were injected into the flank subcutaneously. Mice were randomly assigned (*n* = 5/group) to four groups: Vector, Vector + Cisplatin, hsa-circ-0001030, and hsa-circ-0001030 + Cisplatin. Tumor dimensions were measured with calipers every three days, and tumor volume was calculated as V = (L × W^2^) × 0.5. Starting on day 7 post-implantation, cisplatin 10 mg/kg was administered for a total of eight injections every three days intraperitoneally. At the endpoint, tumors were excised, photographed, weighed, and processed for histology.

### Statistical analysis

All experiments were independently repeated at least three times, and data are presented as the mean ± SD. Statistical analyses were performed using Statistical Package for the Social Sciences (SPSS) software (version 22.0; IBM Corp., Armonk, NY, USA) and GraphPad Prism (version 8.0; GraphPad Software, San Diego, CA, USA). Differences between the two groups were evaluated using Student’s *t*-test, whereas one-way analysis of variance (ANOVA) followed by Tukey’s post hoc test was applied for multiple-group comparisons. Nonparametric data were analyzed using the Mann–Whitney *U* test. Pearson’s correlation analysis was conducted to assess relationships between variables, and Kaplan–Meier survival analysis with the log-rank test was used for patient survival comparisons when applicable. A *P*-value < 0.05 was considered statistically significant. All statistical tests were two-tailed. ^*^P < 0.05; ^**^P < 0.01; ^***^P < 0.001.

## RESULTS

### Identification and characterization of hsa-circ-0001030 as a downregulated and stable cytoplasmic circRNA in TSCC

**Figure 1:**
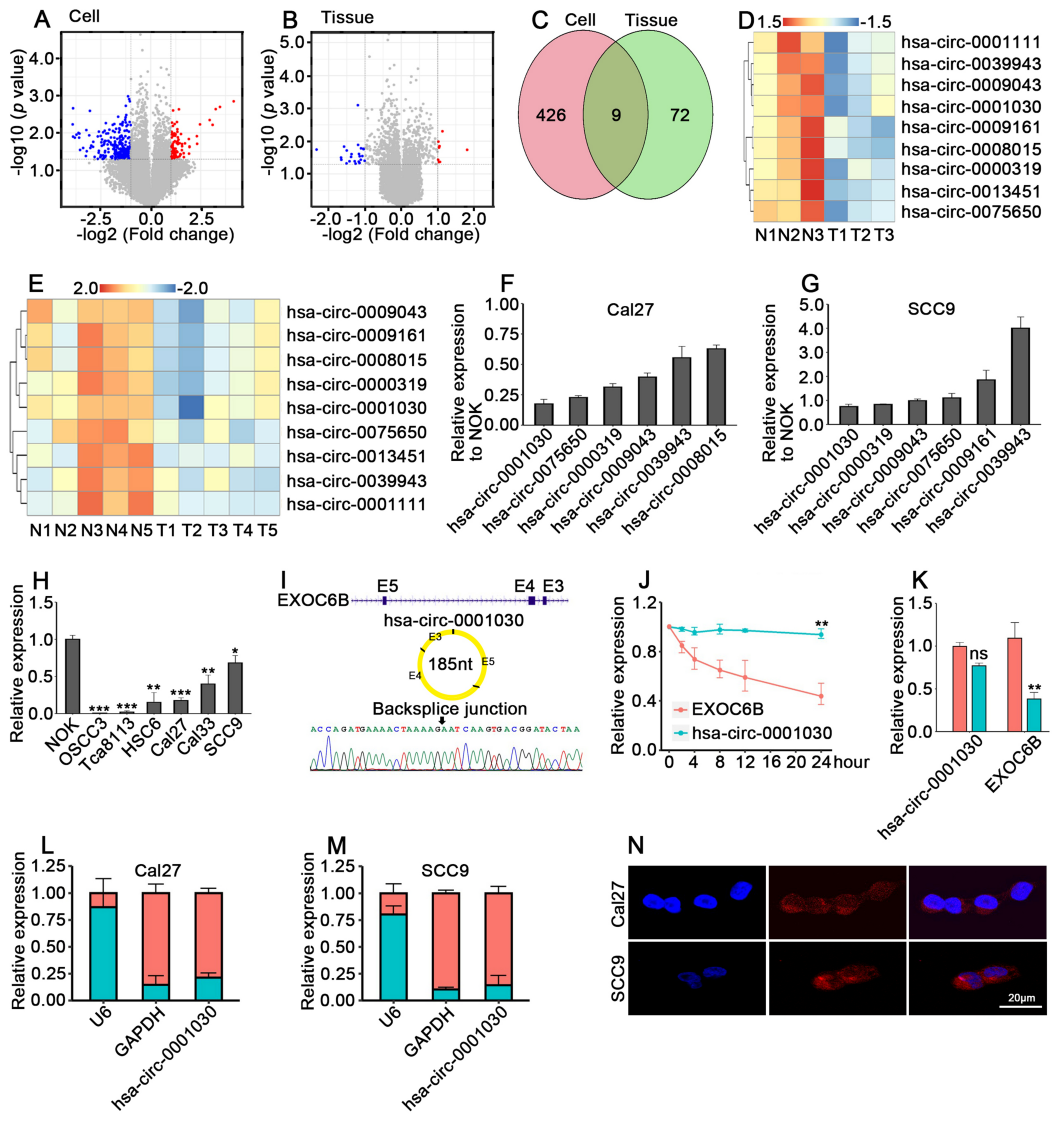
Hsa-circ-0001030 is significantly downregulated and exhibits cytoplasmic localization in TSCC. (A) Volcano plot showing differentially expressed circRNAs between Cal27 and NOK cells; (B) Volcano plot of circRNA expression profiles in five pairs of adjacent normal tissues and TSCC tumor tissues; (C) Venn diagram showing the overlap of downregulated circRNAs identified in cell lines and tissues; (D and E) Top nine downregulated circRNAs in Cal27 *vs.* NOK cells (D) and in TSCC *vs.* adjacent tissues (E); (F and G) qRT-PCR validation of six circRNAs consistently downregulated in both TSCC cells (Cal27, F) and SCC9 cells (G); (H) Relative expression of hsa-circ-0001030 in TSCC cell lines detected by qRT-PCR; (I) The circularization of hsa-circ-0001030 generated by back-splicing of exons 3-5 of the *EXOC6B* gene was illustrated by a schematic diagram; (J) The stability of hsa-circ-0001030 and the parental *EXOC6B* mRNA was assessed by qRT-PCR following actinomycin D treatment; (K) Quantification of hsa-circ-0001030 and *EXOC6B* mRNA expression in cells treated with RNase R or mock control by qRT-PCR; (L and M) Subcellular fractionation analysis showing the distribution of hsa-circ-0001030 in the cytoplasmic and nuclear fractions of Cal27 (L) and SCC9 (M) cells; (N) RNA FISH visualization confirming predominant cytoplasmic localization of hsa-circ-0001030 in Cal27 and SCC9 cells. Data are presented as mean ± SD from at least three independent experiments. *P* < 0.05 was considered statistically significant. ^*^P < 0.05; ^**^P < 0.01; ^***^P < 0.001; ns: not significant. TSCC: Tongue squamous cell carcinoma; circRNAs: circular RNAs; NOK: normal oral keratinocytes; qRT-PCR: quantitative real-time polymerase chain reaction; mRNA: messenger RNA; FISH: fluorescence *in situ* hybridization; SD: standard deviation; GAPDH: glyceraldehyde-3-phosphate dehydrogenase.

To explore the expression landscape of circRNAs in TSCC, we performed circRNA microarray profiling using paired tumor and adjacent normal tissues. Compared with normal tissues, multiple circRNAs were differentially expressed in TSCC, among which a distinct subset showed significant downregulation (|log_2_FC| > 1, *P* < 0.05) [Figure 1A and B]. To identify consistently altered circRNAs in both tissue samples and cell lines, we intersected the two datasets and obtained nine overlapping downregulated circRNAs [Figure 1C]. The expression patterns of these nine candidates are displayed in heatmaps for tissues and cell lines [Figure 1D and E]. Subsequent qRT-PCR validation in Cal27 and SCC9 cells confirmed that six circRNAs were markedly reduced, with hsa-circ-0001030 showing the most significant decrease [Figure 1F and G]. We next examined hsa-circ-0001030 expression across a panel of TSCC cell lines. Consistent with the profiling results, expression of hsa-circ-0001030 was significantly lower in TSCC cells than in NOK [Figure 1H]. The circular structure of hsa-circ-0001030, derived from the *EXOC6B* gene locus (exons 4-5), was validated by divergent primer PCR and Sanger sequencing, which confirmed the characteristic back-splicing junction [Figure 1I]. To assess transcript stability, actinomycin D chase assays were conducted in Cal27 cells. The abundance of hsa-circ-0001030 remained largely unchanged up to 24 h, whereas its linear host transcript EXOC6B decreased over time [Figure 1J]. Similarly, RNase R digestion analysis further proved that hsa-circ-0001030 was resistant to exonuclease degradation, while the linear RNA was completely digested [Figure 1K]. Subcellular fractionation and RNA FISH revealed that hsa-circ-0001030 was predominantly localized in the cytoplasm of Cal27 and SCC9 cells [Figure 1L-N]. Collectively, these results identified hsa-circ-0001030 as a stably expressed, cytoplasmic circRNA which was downregulated in TSCC tissues and cells, suggesting its potential role in regulating TSCC progression.

### Hsa-circ-0001030 suppresses proliferation, migration, and cisplatin resistance of TSCC cells *in vitro*

**Figure 2:**
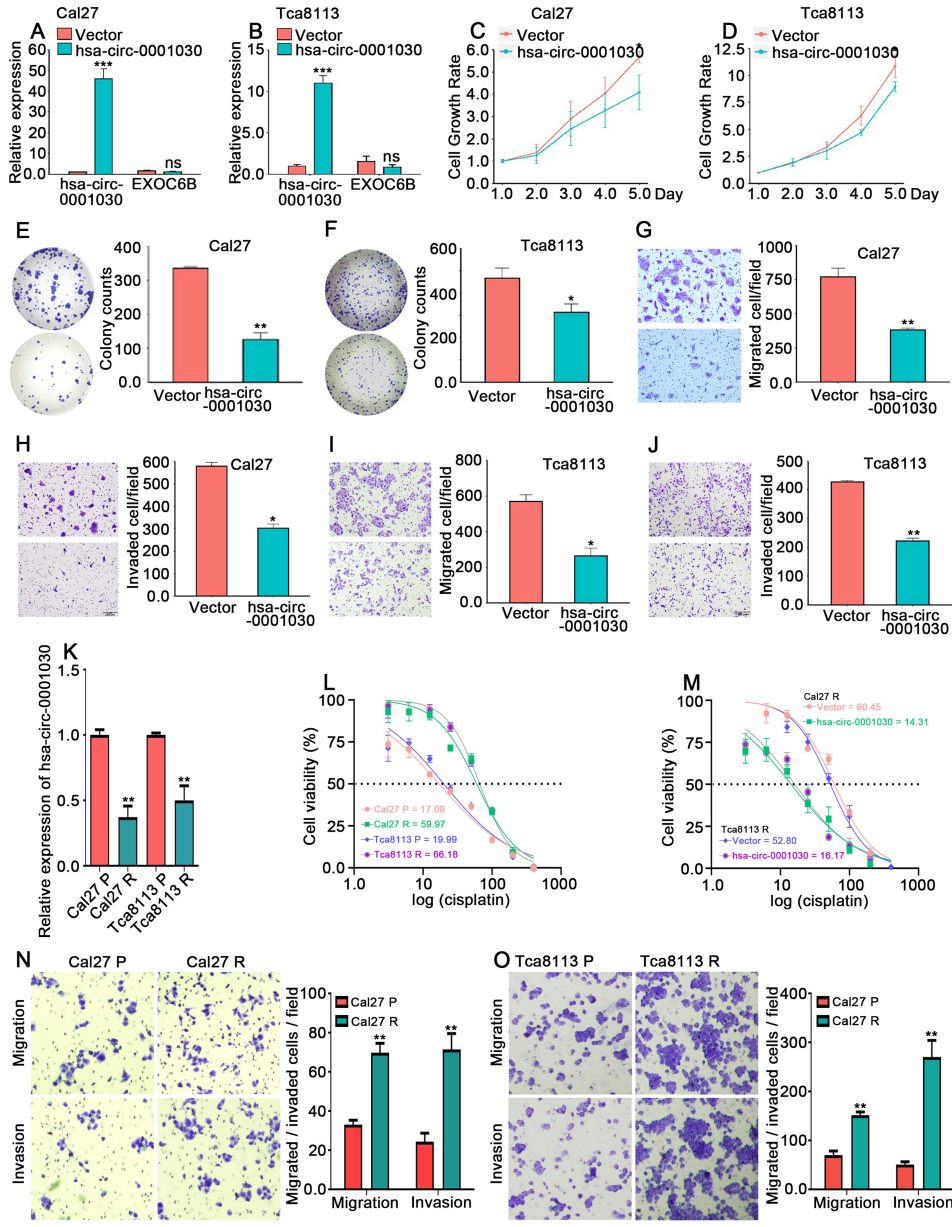
Hsa-circ-0001030 suppresses proliferation, migration, and cisplatin resistance in TSCC cells. (A and B) Expression of hsa-circ-0001030 and the parental *EXOC6B* mRNA in Cal27 cells (A) and Tca8113 (B) cells quantified by qRT-PCR analysis following lentiviral overexpression; (C and D) Cell proliferation of Cal27 cells (C) and Tca8113 cells (D) transfected with vector or hsa-circ-0001030 lentivirus, determined by CCK-8 assay; (E and F) Colony formation assays showing reduced clonogenic growth in Cal27 (E) and Tca8113 (F) cells overexpressing hsa-circ-0001030; (G-J) Representative images and quantification of Transwell assays demonstrating that hsa-circ-0001030 overexpression suppresses migration (G, I) and invasion (H, J) of Cal27 and Tca8113 cells; (K) qRT-PCR analysis showing the relative expression levels of hsa-circ-0001030 in parental (Cal27P, Tca8113P) and cisplatin-resistant (Cal27R, Tca8113R) cell lines; (L and M) Measurement of cisplatin IC_50_ values in Cal27P, Cal27R, Tca8113P, and Tca8113R cells using an MTT assay; (N and O) Representative Transwell images showing enhanced migratory and invasive abilities of cisplatin-resistant Cal27R (N) and Tca8113R (O) cells compared with their parental counterparts. Data are presented as mean ± SD from at least three independent experiments. *P* < 0.05 was considered statistically significant. ^*^P < 0.05; ^**^P < 0.01; ^***^P < 0.001; ns: not significant. TSCC: Tongue squamous cell carcinoma; mRNA: messenger RNA; qRT-PCR: quantitative real-time polymerase chain reaction; CCK-8: cell counting kit-8; IC_50_: half-maximal inhibitory concentration; MTT: 3-(4,5-dimethylthiazol-2-yl)-2,5-diphenyltetrazolium bromide; SD: standard deviation.

To determine the biological significance of hsa-circ-0001030 in TSCC, stably overexpressing hsa-circ-0001030 TSCC cells were established using lentiviral transfection. qRT-PCR analysis confirmed that lentiviral transduction efficiently increased the expression of hsa-circ-0001030 without affecting the parental linear transcript EXOC6B mRNA [Figure 2A and B]. Functional assays demonstrated that hsa-circ-0001030 overexpression inhibited the proliferation of both Cal27 and Tca8113 cells, as evidenced by MTT and CCK-8 assays [Figure 2C and D], and significantly reduced clonogenic capacity in colony formation assays [Figure 2E and F]. Moreover, Transwell assays showed that overexpressing hsa-circ-0001030 significantly decreased the migratory and invasive abilities of TSCC cells [Figure 2G-J]. Given previous findings that circRNAs can modulate chemotherapy sensitivity in TSCC, we next assessed the association between hsa-circ-0001030 expression and cisplatin resistance. Compared with their parental counterparts (Cal27P, Tca8113P), the cisplatin-resistant cells (Cal27R and Tca8113R) exhibited lower hsa-circ-0001030 expression [Figure 2K] and markedly higher IC_50_ values for cisplatin [Figure 2L]. Importantly, hsa-circ-0001030 overexpression restored cisplatin sensitivity in both resistant cell lines [Figure 2M]. Furthermore, Transwell assays revealed that Cal27R [Figure 2N] and Tca8113R [Figure 2O] cells displayed significantly enhanced migratory and invasive capabilities relative to parental cells. Collectively, these findings suggest that hsa-circ-0001030 suppresses TSCC cell proliferation, migration, invasion, and chemoresistance, implicating hsa-circ-0001030 as a potential regulator of cisplatin sensitivity.

### Hsa-circ-0001030 suppresses TSCC tumor growth, metastasis, and enhances cisplatin sensitivity *in vivo*

**Figure 3:**
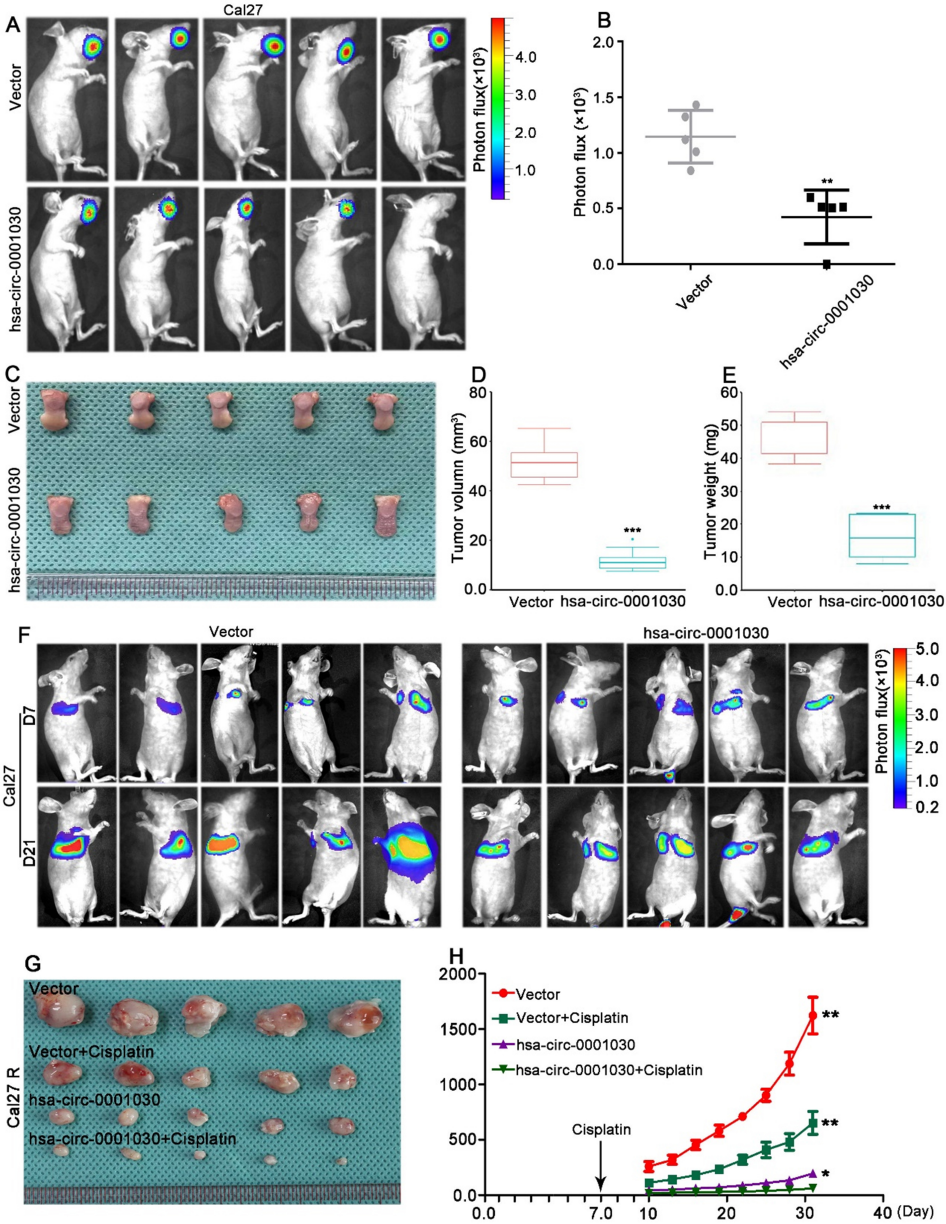
Hsa-circ-0001030 suppresses TSCC tumor growth, metastasis, and enhances cisplatin sensitivity *in vivo*. (A) Representative bioluminescence images of orthotopic tongue xenografts derived from Cal27 cells stably overexpressing hsa-circ-0001030 or vector control (*n* = 5 per group); (B) Quantification of tumor radiance intensity in each group; (C) Representative photographs of excised tumors from the xenograft model; (D and E) Statistical analysis of tumor volume (D) and tumor weight (E) showing that hsa-circ-0001030 overexpression significantly suppressed tumor growth compared with the control group; (F) Representative *in vivo* bioluminescence images of the lung metastasis model (*n* = 5), indicating decreased metastatic signal intensity in the hsa-circ-0001030–overexpressing group; (G) Representative images of subcutaneous xenografts established with cisplatin-resistant Cal27R cells stably overexpressing hsa-circ-0001030; (H) Statistical analysis of tumor volume in Cal27R xenografts treated with cisplatin, demonstrating that hsa-circ-0001030 overexpression enhanced the antitumor efficacy of cisplatin *in vivo*. Data are presented as mean ± SD from five mice per group or three independent experiments. ^*^P < 0.05; ^**^P < 0.01; ^***^P < 0.001. TSCC: Tongue squamous cell carcinoma; SD: standard deviation.

For tumor-suppressive role validation of hsa-circ-0001030 *in vivo*, an orthotopic TSCC model was established using Cal27 cells overexpressing hsa-circ-0001030 stably. Compared with the control (Vector) group, overexpression of hsa-circ-0001030 significantly inhibited tumor growth, as reflected by lesser tumor volumes and reduced bioluminescence signal intensity [Figure 3A and B]. Excised tumors from xenografted mice confirmed that both the tumor weight and size were significantly decreased in the hsa-circ-0001030 overexpression group compared with controls [Figure 3C-E]. In a lung metastasis model, mice injected with hsa-circ-0001030–overexpressing Cal27 cells exhibited distinctly lower fluorescence intensity on *in vivo* imaging [Figure 3F and Supplementary Figure 1A] and fewer and smaller metastatic nodules than those in the Vector group [Supplementary Figure 1B]. Furthermore, in the subcutaneous xenograft model, hsa-circ-0001030 overexpression inhibited tumor progression and enhanced the antitumor efficacy of cisplatin treatment [Figure 3G and H]. Collectively, the *in vivo* results above might demonstrate that hsa-circ-0001030 exerts a tumor-suppressive effect *in vivo* by inhibiting TSCC growth and metastasis, while increasing tumor sensitivity to cisplatin chemotherapy.

### Hsa-circ-0001030 directly binds to PKM2 and inhibits aerobic glycolysis in TSCC cells

**Figure 4:**
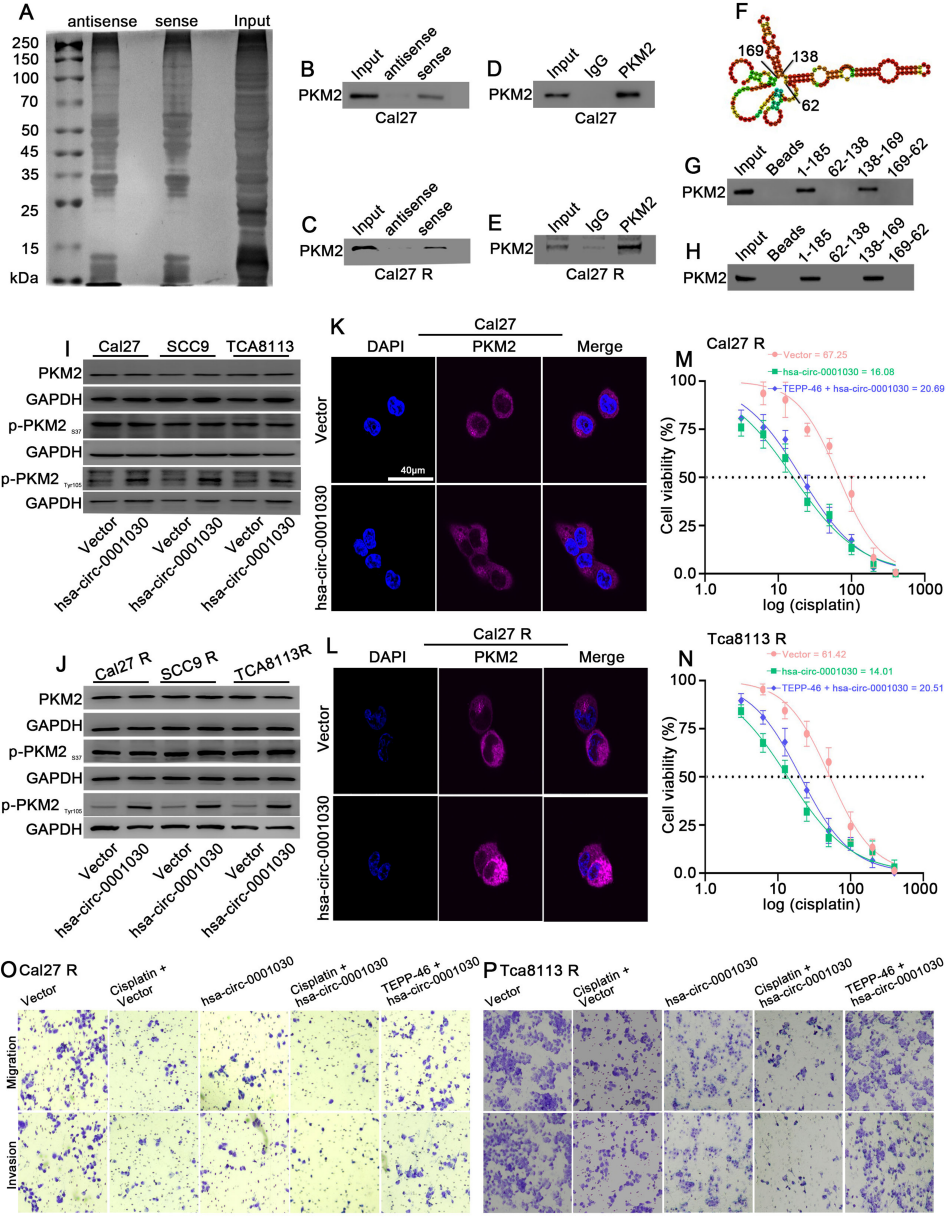
Hsa-circ-0001030 directly binds to PKM2 and inhibits its glycolytic function in TSCC cells. (A) RNA pulldown assays were performed using biotin-labeled specific and non-specific probes for hsa-circ-0001030, and the retrieved proteins were separated by SDS–PAGE; (B and C) Marked enrichment of PKM2 in Cal27 and Cal27R cell lysates pulled down with the hsa-circ-0001030–specific probe was shown by Western blot analysis; (D and E) RIP assays confirming the interaction between PKM2 and hsa-circ-0001030 in Cal27 and Cal27R cells using anti-PKM2 antibody compared with IgG control; (F) Predicted secondary structure of hsa-circ-0001030 based on MFE was generated by the RNAfold WebServer; (G and H) Segmented RNA pulldown analysis identifying the 138-169 nt region of hsa-circ-0001030 as the binding site for PKM2; (I and J) Western blot detection of PKM2 phosphorylation status showing that hsa-circ-0001030 overexpression increases Tyr105 phosphorylation without affecting Ser37 phosphorylation; (K and L) Immunofluorescence analysis showing that hsa-circ-0001030 overexpression prevents PKM2 nuclear translocation, with PKM2 predominantly localized in the cytoplasm; (M and N) MTT assays assessing cisplatin IC_50_ values in hsa-circ-0001030–overexpressing Cal27R and Tca8113R cells treated with the PKM2 agonist TEPP-46, showing enhanced cisplatin sensitivity; (O and P) Representative Transwell images demonstrating that hsa-circ-0001030 overexpression suppresses migration and invasion of Cal27R and Tca8113R cells, whereas treatment with the PKM2 agonist TEPP-46 partially reverses these effects. Data are presented as mean ± SD from three independent experiments. PKM2: Pyruvate kinase M2; TSCC: tongue squamous cell carcinoma; SDS–PAGE: sodium dodecyl sulfate–polyacrylamide gel electrophoresis; RIP: RNA-binding protein immunoprecipitation; MFE: minimum free energy; MTT: 3-(4,5-dimethylthiazol-2-yl)-2,5-diphenyltetrazolium bromide; IC_50_: half-maximal inhibitory concentration; SD: standard deviation; GAPDH: glyceraldehyde-3-phosphate dehydrogenase; DAPI: 4′,6-diamidino-2-phenylindole.

To identify protein partners of hsa-circ-0001030, RNA pulldown assays were performed using a biotin-labeled hsa-circ-0001030 probe. SDS–PAGE analysis of the retrieved proteins revealed distinct bands between 50 and 70 kDa [Figure 4A]. The specificity of probe binding to hsa-circ-0001030 was verified by qRT-PCR [Supplementary Figure 2A and B]. Mass spectrometry identified pyruvate kinase M (PKM) as the top candidate among the pulled-down proteins. Given that PKM has two isoforms, PKM1 and PKM2, and PKM2 plays a predominant role in tumor metabolism, we hypothesized that hsa-circ-0001030 interacts specifically with PKM2. Western blotting of RNA pulldown products confirmed the presence of PKM2 in the hsa-circ-0001030–bound fraction [Figure 4B and C], and RNA immunoprecipitation (RIP) assays further validated the direct binding between PKM2 and hsa-circ-0001030 [Figure 4D and E, Supplementary Figure 2C and D]. To pinpoint the binding region, RNAfold WebServer was introduced to predict the secondary structure of hsa-circ-0001030 based on minimum free energy (MFE) [Figure 4F]. Because RNA–protein interactions often occur in stem-loop regions, the RNA was divided into three segments (62-138 nt, 138-169 nt, and 169-62 nt). After *in vitro* transcription and biotin labeling, RNA pulldown followed by western blotting revealed that the 138-169 nt fragment was responsible for PKM2 binding [Figure 4G and H]. To explore the biological implications of this interaction, GSEA and GO analyses were performed on transcriptome data from hsa-circ-0001030–overexpressing and control cells. Functional enrichment indicated alterations in cellular metabolic pathways, with GSEA showing a significant inhibition of glycolysis (NES = -2.21, FDR < 0.01) upon hsa-circ-0001030 overexpression [Supplementary Figure 2E and F]. Consistent with prior studies demonstrating that nuclear translocation of phosphorylated PKM2 enhances glycolysis and promotes the Warburg effect, we examined PKM2 phosphorylation and localization. It was revealed by Western blot analysis that hsa-circ-0001030 overexpression did not affect PKM2 phosphorylation at Ser37 but increased phosphorylation at Tyr105 [Figure 4I and J], while immunofluorescence assays showed that PKM2 remained cytoplasmic and did not translocate to the nucleus [Figure 4K and L]. We next assessed the functional consequences of this regulation. PKM2 enzymatic activity was lower in cisplatin-sensitive cells (Cal27P, Tca8113P) than in cisplatin-resistant cells (Cal27R, Tca8113R) [Supplementary Figure 2G]. Importantly, overexpression of hsa-circ-0001030 inhibited PKM2 activity [Supplementary Figure 2H and I]. Using glutaraldehyde cross-linking followed by Western blot to examine the oligomeric status of PKM2 after hsa-circ-0001030 overexpression, the results indicated that the proportion of tetrameric PKM2 (~232 kDa) was markedly decreased in both Cal27 and Cal27R cells upon overexpressing hsa-circ-0001030, while the total PKM2 protein level (assessed under non-cross-linking conditions) remained unchanged [Supplementary Figure 2J]. This conformational change was consistent with the observed increase in PKM2 Tyr105 phosphorylation. In addition, we found that overexpression of hsa-circ-0001030 markedly reduced glucose uptake [Supplementary Figure 2K and L] and decreased lactate production [Supplementary Figure 2M and N] in both cisplatin-sensitive and resistant TSCC cells, whereas treatment with the PKM2 agonist TEPP-46 partially reversed these effects. Functionally, MTT assays demonstrated that treatment with the PKM2 agonist TEPP-46 partially reversed the increase in cisplatin sensitivity induced by hsa-circ-0001030 overexpression [Figure 4M and N]. Similarly, Transwell assays revealed that overexpressing hsa-circ-0001030 suppressed migration and invasion in Cal27R [Figure 4O, Supplementary Figure 2O] and Tca8113R [Figure 4P, Supplementary Figure 2P] cells, effects that were partially rescued by TEPP-46 treatment. Collectively, these findings suggest that hsa-circ-0001030 directly binds to PKM2, thereby inhibiting its enzymatic activity, suppressing glycolysis, and attenuating TSCC cell invasion, migration, and chemoresistance.

### Low hsa-circ-0001030 expression is associated with higher malignancy and poor prognosis in TSCC patients

**Figure 5:**
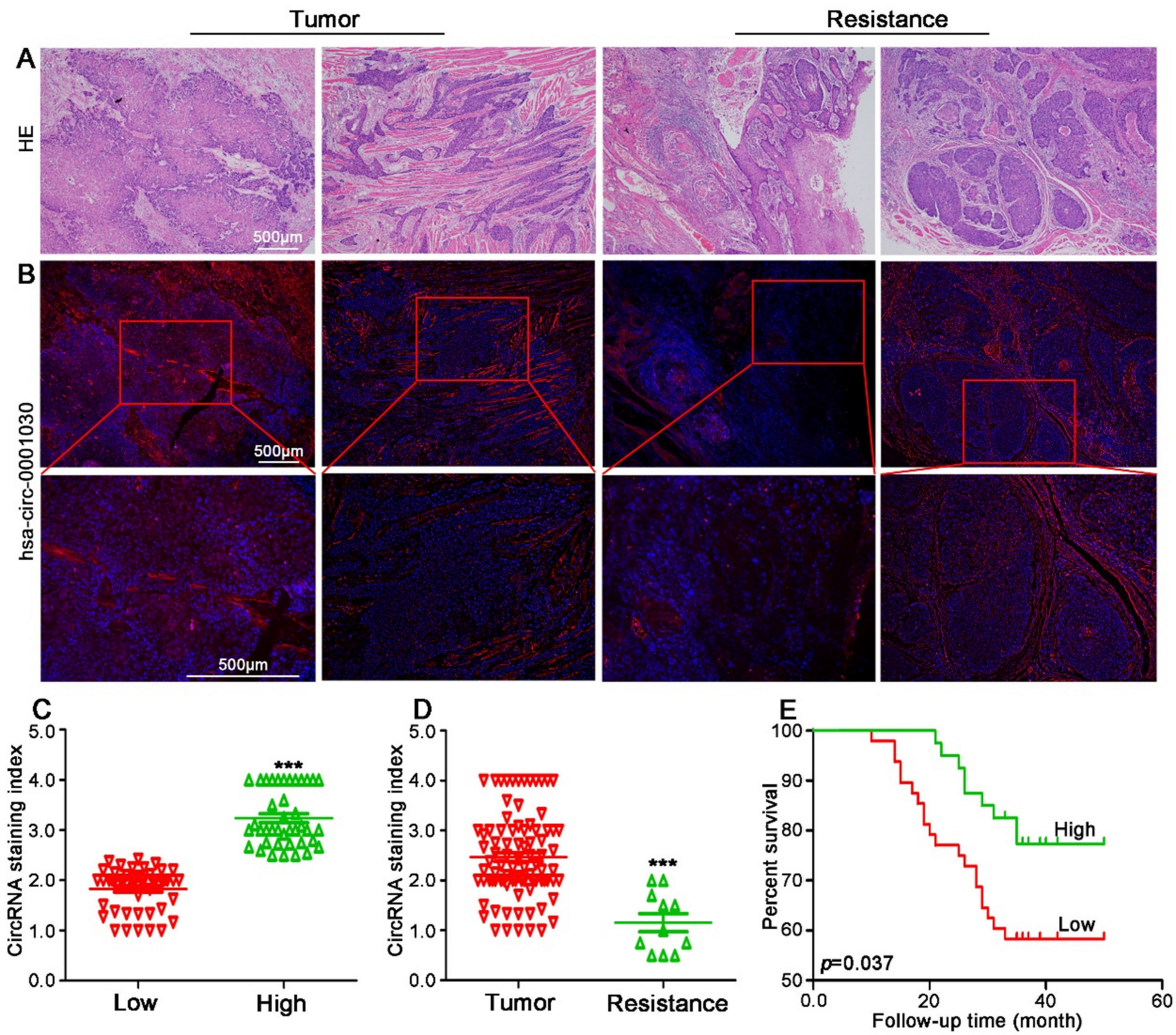
Low expression of hsa-circ-0001030 is associated with chemoresistance and unfavorable prognosis in TSCC patients. (A) Representative HE staining images showing the histopathological morphology of TSCC and adjacent non-tumor tissues; (B) RNA FISH detection of hsa-circ-0001030 expression and subcellular localization in tumor and para-tumor tissues, showing lower cytoplasmic expression in tumor cells; (C) Classification of 88 TSCC patients into high and low hsa-circ-0001030 expression groups based on RNA FISH signal scores (cutoff = 2.46); (D) Comparison of hsa-circ-0001030 expression between the primary tumor group and the cisplatin-resistant tumor group, showing significantly lower expression in the resistant group; (E) Kaplan–Meier survival analysis demonstrating that low hsa-circ-0001030 expressing patients exhibit significantly worse overall survival compared with patients with high expression levels. ^***^P < 0.001. TSCC: Tongue squamous cell carcinoma; HE: hematoxylin–eosin; FISH: fluorescence *in situ* hybridization; circRNA: circular RNA.

To explore the clinical relevance of hsa-circ-0001030 in TSCC, primary tumor specimens from 88 patients and cisplatin-resistant tissues from 11 patients were analyzed. HE staining was first performed to assess histopathological characteristics of TSCC tissues [Figure 5A]. RNA FISH revealed that expression of hsa-circ-0001030 was markedly lower in tumor epithelial regions compared with surrounding stromal tissues and was localized predominantly in the cytoplasm [Figure 5B]. RNA FISH scoring was conducted for all 88 TSCC samples, yielding an average score of 2.46. Patients were therefore categorized into high (≥ 2.46, *n* = 40) and low (< 2.46, *n* = 48) hsa-circ-0001030 expression groups [Figure 5C]. Notably, the cisplatin-resistant tumor group exhibited significantly lower hsa-circ-0001030 scores than the primary tumor group [Figure 5D]. Kaplan–Meier survival analysis demonstrated that low hsa-circ-0001030 expressing patients had a significantly worse overall survival within 50 months compared with the high-expression group [Figure 5E]. These findings suggest that reduced hsa-circ-0001030 expression is associated with unfavorable prognosis in TSCC. Clinicopathological correlation analysis further indicated that expression of hsa-circ-0001030 was not significantly associated with gender or age, but showed a negative correlation with tumor grade and tumor-node-metastasis stage, implying that downregulation of hsa-circ-0001030 reflects advanced disease and higher malignancy [Supplementary Table 2]. These findings suggested that hsa-circ-0001030 downregulation was linked to advanced disease, higher malignancy, and unfavorable prognosis in TSCC.

## DISCUSSION

In the present study, a novel circRNA hsa-circ-0001030 was identified. Hsa-circ-0001030 is significantly downregulated in TSCC and functions as a potent suppressor of tumor progression and cisplatin resistance. Both *in vitro* and *in vivo* assays consistently demonstrated that ectopic expression of hsa-circ-0001030 markedly inhibited TSCC cell proliferation, migration, and invasion, while enhancing the cytotoxic effects of cisplatin. Mechanistically, hsa-circ-0001030 directly bound to PKM2 at the 138-169 nucleotide (nt) region, thereby reducing its enzymatic activity and glycolytic flux without altering PKM2 expression or nuclear translocation. Notably, hsa-circ-0001030 increased phosphorylation of PKM2 at Tyr105, a modification known to restrain the formation of active PKM2 tetramers, resulting in decreased glucose uptake and lactate production. These findings highlight a previously unrecognized circRNA-mediated mechanism regulating the metabolic plasticity of TSCC cells and provide a molecular explanation for how metabolic reprogramming contributes to chemotherapy resistance.

Pyruvate kinase (PK) exists in two isoforms: PKM1 and PKM2. These isoforms were generated by alternative splicing of the *PKM* gene. PKM1 is constitutively active and predominantly expressed in terminally differentiated tissues such as skeletal muscle and brain, where it continuously drives glycolytic flux to support energy production. In contrast, PKM2 is preferentially expressed in malignant and proliferating cells, functioning as a cancer-specific isoform that confers metabolic flexibility and facilitates the Warburg effect, a hallmark of tumor metabolism recognized by enhanced aerobic glycolysis even under normoxic conditions^[13]^. Unlike PKM1, PKM2 can dynamically switch between active tetramers and less active dimers, thereby redirecting glucose metabolites toward biosynthetic and redox-balancing pathways to sustain rapid tumor growth^[14]^. Moreover, PKM2 has non-metabolic nuclear functions, acting as a transcriptional coactivator or protein kinase to regulate oncogenic signaling^[15]^. Because of the unique structural and regulatory properties mentioned above, PKM2 has been recognized as the dominant glycolytic isoform in cancer and a critical driver of metabolic reprogramming and therapeutic resistance. Therefore, our study focused on PKM2 rather than PKM1 to elucidate the circRNA-mediated modulation of tumor metabolism and cisplatin sensitivity in TSCC.

Consistent with reports that PKM2 activation drives cisplatin tolerance in several malignancies, our results indicate that repression of PKM2 activity by hsa-circ-0001030 restores chemosensitivity, underscoring the potential of PKM2 as a therapeutic target to overcome metabolism-driven drug resistance in TSCC^[16,17]^. Here, we demonstrated that hsa-circ-0001030 directly interacts with PKM2, regulating its activity and thereby inhibiting lactate production and glucose uptake in TSCC cells. GSEA analysis further confirmed that hsa-circ-0001030 inhibited the glycolytic pathway.

Various post-translational modifications (PTMs) of PKM2, such as lactylation, phosphorylation, and acetylation, have been identified and shown to alter PKM2 structure as well as its enzymatic or protein kinase activity^[18,19]^. Changes in PKM2 expression also significantly affect tumor development. Similar PKM2-regulating patterns have been observed in non-coding RNA-mediated mechanisms. For instance, long non-coding RNA highly upregulated in liver cancer (lncRNA HULC) enhances the phosphorylation of lactate dehydrogenase A (LDHA) and PKM2 by serving as an adaptor molecule, thereby promoting glycolysis^[20]^. LncRNA AC020978 maintains PKM2 protein stability through proteasomal degradation mediated by ubiquitin, influencing protein level PKM2 expression during non-small cell lung cancer proliferation and glucose metabolism^[21]^. Under hypoxic conditions, circMAT2B enhances glycolysis and promotes HCC progression by activating the circMAT2B/miR-338-3p/PKM2 axis^[11]^. In the present study, hsa-circ-0001030 regulated PKM2 by direct binding and thereby inhibited aerobic glycolysis, implying that the physical interaction between hsa-circ-0001030 and PKM2 affects PKM2’s biological function.

Given that changes in PKM2 expression or phosphorylation may lead to PKM2 nuclear translocation and thereby influence downstream transcription factors such as nuclear factor kappa B (NF-κB), hypoxia-inducible factor-1 alpha (HIF-1α), and signal transducer and activator of transcription 3 (STAT3) in tumors^[14,21,22]^. We further examined PKM2 mRNA and protein levels after hsa-circ-0001030 overexpression. Our results showed that hsa-circ-0001030 overexpression did not affect PKM2 transcription or its nuclear localization, confirming that the changes in aerobic glycolysis were caused by the binding of hsa-circ-0001030 to PKM2 rather than by transcriptional or translational modulation.

PKM2 was further confirmed to directly bind to the 138-169 nt region of hsa-circ-0001030. Based on previous findings indicating that phosphorylation at the PKM2 Ser37 site facilitates its nuclear translocation^[23-25]^, we investigated whether PKM2 (Ser37) phosphorylation occurred in this context. Our results demonstrated that overexpression of hsa-circ-0001030 neither affected PKM2 phosphorylation at Ser37 nor promoted PKM2 nuclear translocation. Similar to our findings, previous research reported that the interaction between circP4HB and PKM2 in lung adenocarcinoma did not alter PKM2 phosphorylation at Tyr105. Although silencing circP4HB reduced PKM2 protein expression, overexpression of circP4HB did not increase PKM2 protein levels but rather enhanced its enzymatic activity by promoting PKM2 tetramer formation, thereby increasing tumor cell glycolysis and proliferation^[26]^. In colorectal cancer, FEZ family zinc finger 1 antisense RNA 1 (FEZF1-AS1) binding stabilized PKM2 protein, leading to an elevated PKM2 expression level in both the nucleus and cytoplasm. Elevated cytoplasmic PKM2 level promoted aerobic glycolysis (PK activity and lactate production), whereas nuclear PKM2 activated the STAT3 signaling pathway, ultimately enhancing tumor proliferation and metastasis^[27]^. Collectively, these studies demonstrate that PKM2 serves as a key effector molecule for non-coding RNA–mediated regulation of aerobic glycolysis, converting phosphoenolpyruvate to pyruvate and driving metabolic reprogramming. Importantly, phosphorylation at the Tyr105 site plays a pivotal role in modulating PKM2 enzymatic activity^[28]^.

Consistent with these reports, our data show that hsa-circ-0001030 overexpression significantly increased PKM2 Tyr105 phosphorylation, leading to decreased PKM2 activity, reduced lactate production, and lower glucose consumption. Phosphorylation at Tyr105 has been shown to restrict PKM2 tetramer formation (its active enzymatic form), although some studies suggest that PKM2 tetramers can form independently of this modification^[25,26]^. In addition, recent evidence highlights that lactylation and acetylation modifications also influence PKM2 tetramer assembly and activity^[29-31]^.

Interestingly, PKM2 phosphorylation and activity changes play critical roles in chemotherapy resistance. Studies have demonstrated that PKM2 phosphorylation can activate specific signaling pathways that promote tumor cell survival and proliferation, thereby enhancing resistance to chemotherapeutic drugs^[32]^. In non-small-cell lung cancer (NSCLC), PKM2 activity is closely linked to cisplatin resistance; inhibiting PKM2 increases cancer cell sensitivity to cisplatin^[33]^. For example, the PKM2 inhibitor shikonin suppresses PKM2 activity, thereby enhancing cisplatin-induced cytotoxicity^[34]^. Together, these findings suggest that PKM2 phosphorylation and activity regulation directly influence tumor cell resistance to cisplatin, and targeting PKM2 represents a promising strategy to overcome metabolic adaptation and drug tolerance.

In summary, our study identifies hsa-circ-0001030, derived from exons 3-5 of the *EXOC6B* gene, as a novel circRNA that suppresses TSCC progression and cisplatin resistance. Both *in vitro* and *in vivo* results demonstrate that hsa-circ-0001030 inhibits TSCC proliferation, invasion, and migration while increasing cisplatin sensitivity. Mechanistically, hsa-circ-0001030 directly interacts with PKM2, inhibits its enzymatic activity, and suppresses glycolytic metabolism. Clinically, low expression of hsa-circ-0001030 correlates with advanced stage, poor differentiation, and unfavorable prognosis in TSCC patients. Collectively, these findings reveal a circRNA–PKM2–glycolysis axis that contributes to chemoresistance, and highlight hsa-circ-0001030 as a potential biomarker and therapeutic target for metabolic intervention in TSCC. Further investigations are warranted to validate the efficacy, delivery, and clinical safety of targeting hsa-circ-0001030 in future translational applications.

Despite the comprehensive design and multiple lines of evidence presented, several limitations remain non-negligible. First, although the *in vitro* and *in vivo* data consistently demonstrate that hsa-circ-0001030 suppresses PKM2 activity and reverses cisplatin resistance, the sample size of the patients remains relatively modest and was acquired from a single institution. Larger multicenter cohorts are required to validate the prognostic and predictive value of hsa-circ-0001030 in diverse patient populations. Second, this work primarily focused on PKM2-mediated glycolytic regulation, whereas circRNAs often exert pleiotropic functions through interactions with miRNAs, RNA-binding proteins, or chromatin regulators. The possibility that hsa-circ-0001030 modulates additional signaling pathways - such as HIF-1α, STAT3, or NF-κB - cannot be excluded and warrants further exploration. Third, the mechanistic assays relied mainly on overexpression systems, which may not fully recapitulate physiological circRNA abundance. Future studies employing CRISPR/Cas13-based knockdown or conditional knockout mouse models will be essential to confirm the endogenous function of hsa-circ-0001030. Fourth, our investigation was limited to the PKM2 phosphorylation at Tyr105; other PTMs, including lactylation and acetylation, may also participate in PKM2 regulation and merit systematic analysis. Finally, the translational applicability of targeting the circRNA–PKM2 axis for therapeutic intervention has not yet been evaluated. Developing delivery platforms - such as circRNA mimics, antisense oligonucleotides, or nanoparticle-mediated systems - and assessing their safety and efficacy in preclinical models will be critical for the next steps. Collectively, addressing these limitations will strengthen the biological and clinical relevance of our findings, meanwhile progressing the facilitation of hsa-circ-0001030 as a potential biomarker and therapeutic target for overcoming cisplatin resistance in TSCC.

## DECLARATIONS

### Availability of data and materials

The datasets generated and analyzed during the current study are available from the corresponding author upon reasonable request, due to institutional data privacy restrictions.

### Copyright

© The Author(s) 2026.
