# Supplementary material for: Circular RNA hsa-circ-0001030 suppresses proliferation and cisplatin tolerance in TSCC via interaction with PKM2
Source: Cancer Drug Resist. 2026 Jan 7;9:1. doi: 10.20517/cdr.2025.200 (PMC12883343; doi:10.20517/cdr.2025.200)
Supplement: Supplementary file 1 [file cdr-9-1-SupplementaryMaterials.pdf]

Supplementary Materials

Circular RNA hsa-circ-0001030 suppresses proliferation and cisplatin tolerance in TSCC via interaction with PKM2

Haojie Yang[1,2,#], Yingzhe Yan[1,2,#], Zicong Tan[1,2,#], Xiaoying Xu[1,2], Kang Chen[1,2], Qin Li[2,3], Ning Liufu[1,2], Fengtao Ji[1,2]

[1]Department of Anesthesia, Sun Yat-sen Memorial Hospital, Sun Yat-sen University, Guangzhou 510000, Guangdong, China.
[2]Guangdong Provincial Key Laboratory of Malignant Tumor Epigenetics and Gene Regulation, Guangdong-Hong Kong Joint Laboratory for RNA Medicine, Medical Research Center, Sun Yat-sen Memorial Hospital, Sun Yat-sen University, Guangzhou 510000, Guangdong, China.
[3]Department of Pathology, Sun Yat-Sen Memorial Hospital, Sun Yat-Sen University, Guangzhou 510000, Guangdong, China.
[#]These authors contributed equally to this work and share first authorship.

Correspondence to: Prof. Fengtao Ji, Ning Liufu, Department of Anesthesia, Sun Yat-sen Memorial Hospital, Sun Yat-sen University, Guangzhou 510000, Guangdong, China. E-mail: jift@mail.sysu.edu.cn; liufn3@mail.sysu.edu.cn

Supplementary Table 1. Primer sequence

	Forward Sequence (5'-3')	Reverse Sequence (5'-3')
**ACTB**	CATGTACGTTGCTATCCAGGC	CTCCTTAATGTCACGCACGAT
**GAPDH**	TGCACCACCAACTGCTTAGC	GGCATGGACTGTGGTCATGAG
**Hsa-circ-0001030**	AGCAAACTGAGGGACCAGATG	GCTTCAGCTCTTCCATTGCT
**Hsa-circ-0000319**	CAATGTGGAAGGTCCGGAGG	TTGAAGCCAGGCATGCTGAT
**Hsa-circ-0008015**	TCCATCATGTACACAGGAGCAC	TGGTACCGATTGCGACCAGTG
**Hsa-circ-0009043**	GGAGAGCATCCGCAAACATTC	TAGTCGACACTGCTTCAGCTC
**Hsa-circ-0009161**	TCGCAATCGGTACCAAAGGT	CCGATCTGGGAAAGCTGTGAT
**Hsa-circ-0039943**	AGGAGGTGGCTTATGAAAGTGT	GCAGCAAGGCCTAATTCCAC
**Hsa-circ-0075650**	ACTTTGGACGAGAGCTGCAA	CACACCTTGAGCAGAAAGACC
**EXOC6B**	TCCTGCGAGAGATCGAGAGC	CTTCTCCATGAAACGTCCATGT
**U6**	CTCGCTTCGGCAGCACATATACT	ACGCTTCACGAATTTGCGTGTC

Supplemental Table 2. Hsa-circ-0001030 expression and clinicopathological parameters

**Variables**		**Expression**		**Total**	***P* value**
		**Low**	**High**		
**Sex**					0.156
	Male	38	26	64	
	Female	10	14	24	
**Age (year)**					0.83
	≤ 61	28	22	50	
	> 61	20	18	38	
**TNM stage**					0.002
	I / II	36	17	53	
	III /IV	12	23	35	
**Grade**					0.04
	I	11	18	29	
	II/III	37	22	59	

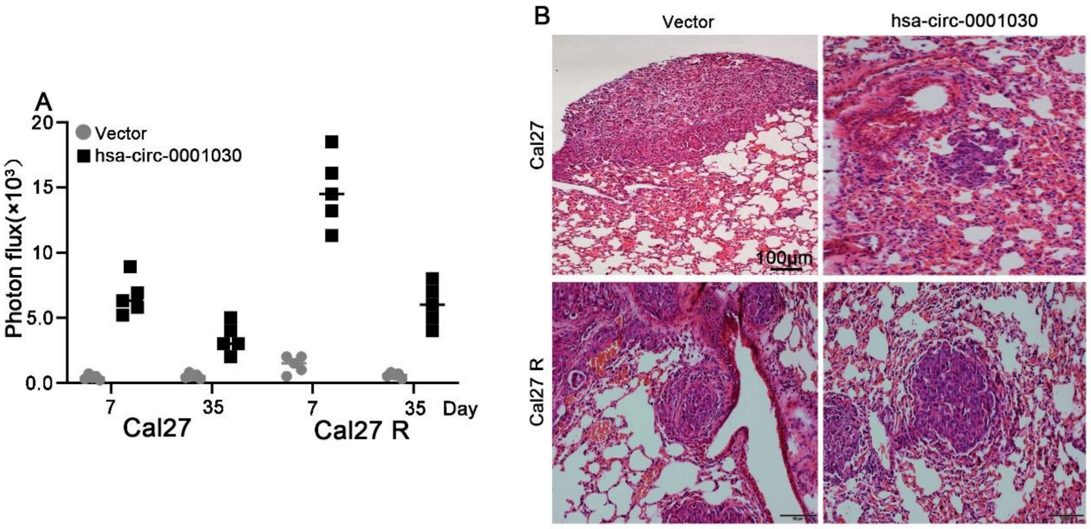

Supplementary Figure 1. Hsa-circ-0001030 inhibits TSCC growth and pulmonary metastasis *in vivo.* (A) Quantitative analysis of bioluminescent radiance intensity in each group of tumor-bearing mice, showing reduced signal intensity in the hsa-circ-0001030 overexpression group compared with the vector control group; (B) Representative hematoxylin–eosin (HE) staining images of lung sections from each group, demonstrating fewer and smaller metastatic nodules in mice overexpressing hsa-circ-0001030.

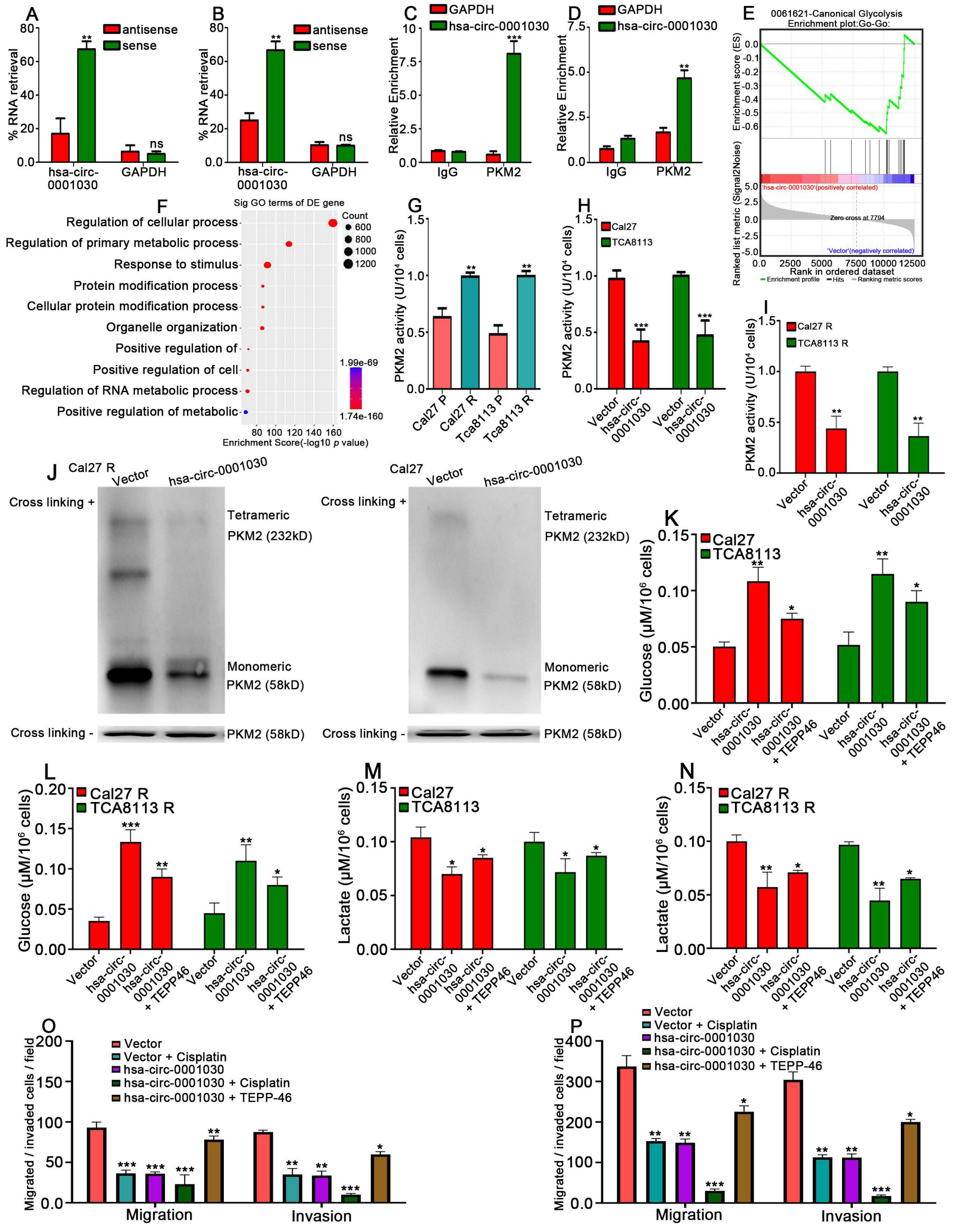

Supplementary Figure 2. Hsa-circ-0001030 directly binds to PKM2 and regulates glycolytic activity in TSCC cells. (A and B) Specific binding of biotin-labeled probes to hsa-circ-0001030 was validated by qRT-PCR, confirming probe specificity; (C and D) RNA immunoprecipitation (RIP) analysis showing significant enrichment of hsa-circ-0001030 in samples immunoprecipitated with anti-PKM2 antibody compared with IgG control; (E) Gene Set Enrichment Analysis (GSEA) of transcriptomic profiles revealing downregulation of the glycolytic pathway in cells overexpressing

hsa-circ-0001030 compared with controls; (F) Gene Ontology (GO) enrichment analysis of differentially expressed genes between the hsa-circ-0001030 overexpression group and the control group, indicating that hsa-circ-0001030 modulates cellular metabolic processes; (G) Enzymatic activity assay of PKM2 among Cal27P, Cal27R, Tca8113P, and Tca8113R cells, showing elevated PKM2 activity in cisplatin-resistant sublines; (H and I) PKM2 activity assays demonstrating that hsa-circ-0001030 overexpression reduces PKM2 enzymatic activity in both parental and resistant TSCC cells; (J) Representative Western blot analysis of PKM2 oligomeric status in Cal27 and Cal27R cells with or without *hsa-circ-0001030* overexpression. Cell lysates were treated with 0.025% glutaraldehyde for cross-linking (+) or left untreated (-) prior to SDS–PAGE. The high-molecular-weight bands (~232 kDa) represent the tetrameric form of PKM2, whereas the 58 kDa bands correspond to monomeric PKM2; (K and L) Glucose consumption assays showing that hsa-circ-0001030 overexpression decreases glucose uptake in TSCC cells; (M and N) Lactate production assays demonstrating that hsa-circ-0001030 overexpression significantly lowers lactate output in TSCC cells; (O and P) Quantitative analysis of Transwell assays confirming that hsa-circ-0001030 overexpression suppresses the migration and invasion abilities of TSCC cells.
